# Rapid induction of autoantibodies during ARDS and septic shock

**DOI:** 10.1186/1479-5876-8-97

**Published:** 2010-10-14

**Authors:** Peter D Burbelo, Nitin Seam, Sandra Groot, Kathryn H Ching, Brian L Han, G Umberto Meduri, Michael J Iadarola, Anthony F Suffredini

**Affiliations:** 1 Neurobiology and Pain Therapeutics Section, Laboratory of Sensory Biology, National Institute of Dental and Craniofacial Research, National Institutes of Health, Bethesda, Maryland 20892, USA; 2 Critical Care Medicine Department, Clinical Center, National Institutes of Health, Bethesda, Maryland 20892, USA; 3 Pulmonary and Critical Care Medicine Department, Veterans Affairs Medical Center, Washington, District of Columbia 20422, USA; 4 Division of Pulmonary, Critical Care, and Sleep Medicine, Veterans Affairs Medical Center, Memphis, Tennessee 38163, USA

## Abstract

**Background:**

Little is known about the induction of humoral responses directed against human autoantigens during acute inflammation. We utilized a highly sensitive antibody profiling technology to study autoantibodies in patients with acute respiratory distress syndrome (ARDS) and severe sepsis, conditions characterized by intensive immune activation leading to multiple organ dysfunction.

**Methods:**

Using Luciferase Immunoprecipitation Systems (LIPS), a cohort of control, ARDS and sepsis patients were tested for antibodies to a panel of autoantigens. Autoantibody titers greater than the mean plus 3 SD of the 24 control samples were used to identify seropositive samples. Available longitudinal samples from different seropositive ARDS and sepsis patient samples, starting from within the first two days after admission to the intensive care, were then analyzed for changes in autoantibody over time.

**Results:**

From screening patient plasma, 57% of ARDS and 46% of septic patients without ARDS demonstrated at least one statistically significant elevated autoantibody compared to the controls. Frequent high titer antibodies were detected against a spectrum of autoantigens including potassium channel regulator, gastric ATPase, glutamic decarboxylase-65 and several cytokines. Analysis of serial samples revealed that several seropositive patients had low autoantibodies at early time points that often rose precipitously and peaked between days 7-14. Further, the use of therapeutic doses of corticosteroids did not diminish the rise in autoantibody titers. In some cases, the patient autoantibody titers remained elevated through the last serum sample collected.

**Conclusion:**

The rapid induction of autoantibodies in ARDS and severe sepsis suggests that ongoing systemic inflammation and associated tissue destruction mediate the break in tolerance against these self proteins.

## Introduction

Serum antibodies are essential components of adaptive immunity, but are also involved in the pathogenesis of many autoimmune diseases. While much is known about the control of host antibody production following pathogen exposure or vaccination [1], the induction of autoantibodies in human autoimmune and other diseases remains poorly defined. In genetically susceptible individuals, infection and other environmental insults have been speculated to trigger immune responses by different mechanisms including induction of cytokines, stimulation of toll-like receptors and other pattern recognition receptors, the release of self antigens by damaged cells and tissues and/or molecular mimicry [2]. However to date, little is known about the spectrum of autoantibody responses and the kinetics of autoantibody induction during acute infection and systemic inflammation.

Acute respiratory distress syndrome (ARDS) and severe sepsis are acute inflammatory conditions associated with high morbidity and mortality, often involving multiple organ failure [3,4]. ARDS is caused by a wide variety of infectious or inflammatory insults to the lung that may occur by direct (e.g. pneumonia) or indirect injury (e.g. peritonitis). The pathologic hallmarks of ARDS are diffuse alveolar damage manifested by disruption of the alveolar-capillary interface, as well as the accumulation of inflammatory cells and protein-rich exudates in the alveolar spaces [4]. Patients with ARDS have elevated levels of inflammatory mediators such as TNF-α, IL-1β, IL-6 and IL-8 in lung lining fluid as well as in the circulation [5]. In sepsis a nidus of infection causes a local and systemic inflammatory response [3]. However as sepsis persists, there is a rapid shift towards an anti-inflammatory immunosuppressive state that likely involves T-cell anergy [6,7], increased anti-inflammatory cytokines [8] and the loss of dendritic cells, B lymphocytes and CD4+ T lymphocytes [9,10].

Luciferase immunoprecipitation systems (LIPS), offers a highly quantitative and sensitive method to measure antibody responses against large numbers of foreign antigens and autoantigens [11-16]. In this study, LIPS was used to profile plasma from patients with ARDS or sepsis against a panel of known autoantigens. Within 10 to 14 days after the onset of illness, nearly 50% of the patients show high antibody titers to at least one autoantigen. Remarkably, analysis of serial samples revealed that the induction of these autoantibodies occurred rapidly, often within 1-7 days after intensive care unit admission and in some cases remained elevated for several weeks. The mechanisms and time course for the rapid induction of autoantibodies seen in ARDS and sepsis may occur in other conditions including autoimmune diseases.

## Methods

### Patient Samples

Plasma samples were obtained from patients and healthy control subjects under institutional review board-approved protocols at the NIH Clinical Center and from the University of Tennessee Health Science Center [17]. Plasma samples were obtained from heparinized venous whole blood by centrifugation and stored in aliquots at -80°C. Plasma samples from the 24 normal volunteers were collected at the NIH Clinical Center, while the 35 ARDS patients were selected from a randomized trial investigating prolonged methylprednisolone treatment in early severe ARDS conducted at the University of Tennessee [17]. The 13 patients with sepsis were selected from a randomized trial investigating prolonged hydrocortisone therapy in severe sepsis not complicated by ARDS, also conducted at the University of Tennessee Health Science Center. These ARDS and sepsis plasma samples were used in this retrospective, exploratory study to examine whether autoantibodies are generated in periods of acute inflammation. Samples were selected from patients whom time points were available at least 7 days into acute inflammatory conditions. We attempted to use samples from patients with positive culture results: all patients in the sepsis cohort had positive culture results, as did 80% of the patients in the ARDS cohort. As part of a clinical protocol, all ARDS and sepsis patients were in the intensive care unit (ICU) and evaluated with a battery of clinical tests including lung injury scores (LIS) and multiple organ dysfunction syndrome (MODS) scores. For the 35 ARDS patients, 22 patients were treated with methylprednisolone and 13 were not treated with steroids. For severe sepsis patients, ten received stress-doses of hydrocortisone, and three did not. The characteristics of the ARDS and sepsis patients are summarized in Table 1 and include age, gender, APACHE 3 scoring, methylprednisolone/hydrocortisone treatment frequency, infection status and in-hospital survival rate. None of the patients had a known history of autoimmune disorders or were receiving outpatient treatment with corticosteroids or other immunosuppressants prior to clinical presentation.

**Table 1:** Clinical Characteristics Based on Autoantibody Status

	Autoantibody Positive*^a ^*(n = 20)	Autoantibody Negative*^a ^*(n = 15)
**ARDS**		

Age yrs (mean ± SD)	45 ± 14	52 ± 16

Gender	8 male (40%)	10 male (67%)

APACHE 3 score (mean ± SD)	58 ± 17	62 ± 16

Methylprednisolone treatment*^b^*	13/20 (65%)	9/15 (60%)

Infections	Gram positive bacteria: 12 Gram negative bacteria: 3 Fungal: 1 Culture negative: 5	Gram positive bacteria: 4 Gram negative Bacteria: 7 Fungal: 1 Viral: 1 Culture negative: 2

In-hospital survival	18/20 (90%)	9/15 (60%)

		

**Severe Sepsis**		

	**Autoantibody Positive***^**a **^***(n = 6)**	**Autoantibody Negative***^**a **^***(n = 7)**

Age yrs (mean ± SD)	54 ± 21	63 ± 18

Gender	5 male (83%)	7 male (100%)

APACHE 3 score (mean ± SD)	75 ± 26	68 ± 23

Hydrocortisone treatment*^c^*	5/6 (83%)	5/7 (71%)

Infections	Gram positive bacteria: 6 Gram negative bacteria: 0	Gram positive bacteria: 3 Gram negative bacteria: 4

In-hospital survival	4/6 (67%)	5/7 (71%)

*^a^*As determined by LIPS.

*^b^*Methylprednisolone dose - 1 mg/kg/day for 14 days then tapered.

*^c^*Hydrocortisone dosage - 300 mg initially then 10 mg per hour for seven days.

### Ruc-antigen fusions and LIPS analysis

Many of the autoantigens used in these LIPS studies including those for glutamic decarboxylase-65 (GAD65), AQP-4, gastric ATPase and a fragment of Ro52 (Ro52-Δ2) have been previously described [14-16]. Four cytokines (Interferon-γ, Interferon-ω, Interleukin-6 and interleukin-1α) corresponding to the processed cytokine missing the signal peptide sequences were also generated as C-terminal *Ruc*-antigen fusions [18]. In addition a new lung autoantigen, KCNRG and four other cytokines were constructed as C-terminal antigen fusions downstream of *Renilla *luciferase (Ruc) using the pREN2 vector [13]. DNA sequencing was used to ensure the integrity of this new construct.

LIPS assay was performed at room temperature as described [19]. In these assays, sera were processed in a 96-well format. A "master plate" was first constructed by diluting patient sera 1:10 in assay buffer A (50 mM Tris, pH 7.5, 100 mM NaCl, 5 mM MgCl_2_, 1% Triton X-100) in a 96-well polypropylene microtiter plate. For evaluating antibody titers by LIPS, 40 μl of buffer A, 10 μl of diluted human sera (1 μl equivalent), and 1 × 10^7 ^light units (LU) of Ruc-antigen Cos1 cell extract, diluted in buffer A to a volume of 50 μl, were added to each well of a polypropylene plate and incubated for 60 minutes at room temperature on a rotary shaker. Next, 5 μl of a 30% suspension of Ultralink protein A/G beads (Pierce Biotechnology, Rockford, IL) in PBS were added to the bottom of each well of a 96-well filter HTS plate (Millipore, Bedford, MA). To this filter plate, the 100 μl antigen-antibody reaction mixture was transferred and incubated for 60 minutes at room temperature on a rotary shaker. The washing steps of the retained protein A/G beads were performed on a Tecan plate washer with a vacuum manifold. After the final wash, LU were measured in a Berthold LB 960 Centro microplate luminometer (Berthold Technologies, Bad Wilbad, Germany) using coelenterazine substrate mix (Promega, Madison, WI). All light unit (LU) data were obtained from the average of two separate experiments and not corrected for negligible background protein A/G bead binding. Patient samples positive at day 10 for ARDS or day 14 for sepsis were reexamined for changes in antibody titers using all available serial samples.

### Statistical analysis

GraphPad Prism software (San Diego, CA) was used for statistical analysis. Due to the overdispersed nature of the autoantibody titers, the healthy control subjects (CTRL) are reported as the geometric mean titer (GMT) ± 95% confidence interval. For determining the cut-off limits for each of the LIPS tests, the mean value of the 24 control samples plus 3 standard deviations (SD) in the first cohort was used and is indicated in the figures. The non-parametric Mann-Whitney *U *test was used for comparison of antibody titers in different groups. Using contingency tables, the Fischer's exact test was used to determine the statistical significance between autoantibody seropositivity and in-hospital survival.

Data transformation and a heatmap were used to visualize the autoantibody profiles of the participants as a single graphic. In order to create this heatmap, the mean and standard deviation of the antibody titers for each antigen in the 24 control samples was first generated as a reference scale. Next, antibody titer values for each antigen-antibody measurement greater than the control mean plus 3 SD were color-coded to signify the relative number of standard deviations above these cut-off values. Lastly, the samples were rank ordered with respect to anti-KCNRG autoantibodies, the most informative autoantigen in the ARDS and sepsis patients.

## Results

### Detection of high titer autoantibodies to proinflammatory cytokines in selected ARDS and sepsis patients

**Figure 1:**
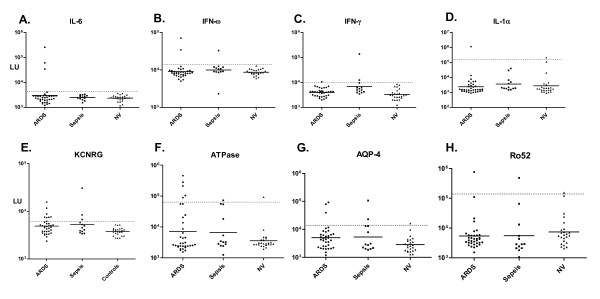
**Autoantibodies in patients with ARDS or severe sepsis**. Shown are results from 24 controls, 35 ARDS and 13 sepsis patients. Each symbol represents a sample from one individual patient. The autoantibody titers for (A) IL-6 (B) IFN-ω, (C) IFN-γ, (D) IL1-α, (E) KCNRG and (F) gastric ATPase, (G) AQP-4 and (H) Ro52 antibody titers are plotted on the Y-axis using a log_10 _scale. The geometric mean antibody titer for the ARDS, sepsis and controls are shown by the short solid lines. The dashed line represents the cut-off level for determining seropositivity and is derived from the mean plus 3 SD of the antibody titer of the 24 controls. *P *values were calculated using the Mann Whitney *U *test and were only significant for anti-KCNRG autoantibodies (control vs. ARDS; *P *= 0.006 and control vs. sepsis; *P *= 0.03).

Based on the hypothesis that anti-cytokine autoantibodies might predispose a patient to infection or inflammation, 24 controls, 35 ARDS and 13 sepsis patients were screened for autoantibodies to a panel of cytokines using LIPS. To increase the likelihood of detecting anti-cytokine antibodies at peak levels, patient samples were analyzed from ARDS at day 14 and sepsis at day 10. Since the normal range of anti-cytokine autoantibody titers is not known, and in order to facilitate the identification of elevated anti-cytokine autoantibodies, a cut-off threshold based on autoantibody titers greater than the mean plus 3 SD of the 24 control samples was used to identify potential seropositive samples. Based on this criterion, a selected number of ARDS and sepsis patients showed autoantibodies against several cytokines that were often 10 to 1,000-fold higher than the GMT of the controls (Figure 1). For example, three ARDS serum samples had high anti-interleukin-6 (IL-6) autoantibody titers with values of 34,213, 60,719, and 255,074 LU, which were all markedly higher than the GMT of anti-IL-6 antibodies in the controls with a value of 2,347 LU [95% confidence interval (CI); 2,080-2,649] (Figure 1A). As shown in Figure 1B, two ARDS samples were positive for anti-interferon-ω (INF-ω) autoantibodies with values of 34,348 and 70,779 LU, while the GMT of the control group was only 8,658 LU (95% CI; 7,993-9,379). Significantly elevated anti-interferon-γ (INF-γ) antibodies were also detected in one ARDS and one septic patient (Figure 1C). Finally, one ARDS sample showed a positive anti-interleukin-1α (IL1-α) antibody titer of 1,136,872 LU, which was above the cut-off derived from the controls (Figure 1D). Testing a number of other cytokines, including interferon-α, BAFF (TNF family member), April (a proliferation-inducing ligand) and IL-12, did not reveal autoantibody positivity in any of the ARDS or sepsis patients (data not shown). Together these results suggest that some ARDS and sepsis patients generate high levels of serum autoantibodies to certain cytokines which might reflect autoimmunization against these particular cytokines seen in these patients.

### Detection of immunoreactivity to diverse autoantigen targets in ARDS and sepsis

In light of detecting anti-cytokine autoantibodies in both ARDS and sepsis patients, other potential autoantigens were also evaluated. Since we hypothesized that ARDS and septic patients might show immunoreactivity with antigens derived from damaged tissue and organs, we tested a panel of known autoantigens associated with several autoimmune diseases. The autoantigens Jo-1, MuSK, and La failed to show any statistically significant responses in both patients with ARDS and those with severe sepsis (data not shown). From screening several other autoantigens, we detected autoantibodies against the lung-specific autoantigen potassium channel regulator (KCNRG). Although the anti-KCNRG autoantibody titers were modestly elevated compared to the anti-cytokine autoantibodies, 23% (8/35) of the ARDS and 25% (3/12) of the sepsis patients had statistically significant autoantibody titers that were higher than the control cut-off (Figure 1E). Mann Whitney *U *test analysis revealed significantly higher detectable anti-KCNRG autoantibody titers in both the ARDS (*P *< 0.006) and sepsis patient groups (*P *< 0.03) compared to the controls (Figure 1E). These results suggest that the KCNRG protein is a target of autoantibodies in patients with ARDS and sepsis.

Screening of several other autoantigens, including gastric ATPase, GAD65, AQP-4 and Ro52 also revealed high titer autoantibodies in several patients from the ARDS and severe sepsis cohorts. For example, elevated anti-gastric ATPase autoantibodies, higher than the cut-off derived from the controls, were found in 14% of the ARDS patients (5/35) as well as one patient with severe sepsis (Figure 1F). Testing for anti-AQP-4 antibodies revealed that 9% (3/35) of the ARDS and 15% (2/13) of sepsis samples had antibody titers above the cut-off value of the mean plus 3 SD of the 24 control samples (Figure 1G). High titer autoantibodies above the control cut-off were also detected to GAD65 in three ARDS and two sepsis patients (data not shown). Lastly, one ARDS and one sepsis patient had statistically significant levels of autoantibodies to Ro52 (Figure 1H). Together, these results suggest that ARDS and sepsis patients have a high frequency of autoantibodies against a number of diverse autoantigen targets that are classically associated with several different autoimmune conditions.

### Autoantibody profiles in ARDS and sepsis

**Figure 2:**
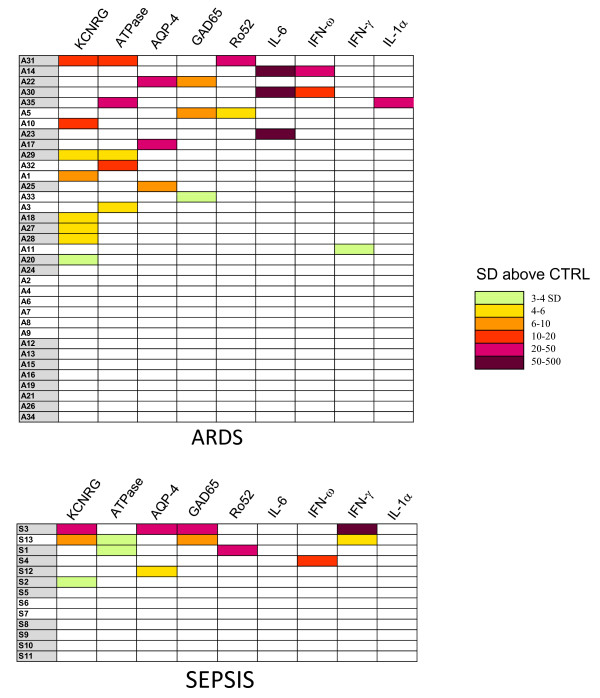
**Heatmap analysis of autoantibody profiles in ARDS and sepsis patients**. Autoantibody titers to the informative autoantigens are shown for each of the 35 ARDS patient and 13 sepsis patients. The titer values greater than the mean of the 24 normal volunteers plus 3 SD were color-coded from green to dark purple to signify the relative number of SD above these reference values. Shaded codes denote patients who received corticosteroids as part of their treatment.

To more easily understand patient immunoreactivity to the different antigens and relative titers, a colored heatmap was employed. For this heatmap, antibody titer values for each antigen-antibody measurement greater than the cut-off of the control mean plus 3 SD were color-coded to signify the relative number of standard deviations above these cut-off values. Analysis of controls revealed that 5 of the normal volunteers showed positive single autoantibody responses in the range of 3-4 SD (data not shown and Figure 1). In contrast, some but not all ARDS and sepsis patients showed heterogeneous immunoreactivity to the autoantigen panel with antibody titers ranging from 3 to 394 SD above the mean of controls (Figure 2). The most frequently positive autoantigen was the KCNRG lung protein, followed by the gastric ATPase, AQP-4, GAD65 neural autoantigens and finally the Ro52 protein (Figure 2). As evident from the heatmap, several of the ARDS and septic shock patients showed positive autoantibody responses to multiple autoantigens. In general, patients showing autoantibodies to multiple targets were patients with the highest autoantibody titers. The most dramatic example of this was a patient with bacterial meningitis (S3) who showed high titer autoantibodies to four different autoantigens including KCNRG, AQP-4, GAD65 and INF-γ (Figure 2). Some of the other patients with high titer anti-cytokine autoantibodies also showed interesting co-profiles: two ARDS patients (A14 and A30) were co-positive for only IL-6 and interferon-ω autoantibodies, one ARDS patient (A35) was co-positive for IL-1α and ATPase autoantibodies and one sepsis patient (S13) with interferon-γ autoantibodies was also positive for anti-KCNRG and anti-GAD65 autoantibodies (Figure 2). Inspection of the heatmap also shows that there was no difference in the prevalence or relative autoantibody in patients treated with and without steroids (Figure 2 and Table 1). Taken together these results highlight the heterogeneity of targets and autoantibody titers seen acutely in ARDS and sepsis patients and suggest that steroid treatment has little or no effect on the production of autoantibodies in these conditions.

Since only 57% of ARDS and 46% of septic patients demonstrated at least one statistically significant elevated autoantibody compared to the controls, at present it is difficult to make any general conclusions about the predictive value of these autoantibodies for determining severity. However, the relationship between short-term survival and autoantibodies was examined. As shown in Table 1, the ARDS autoantibody positive patients showed a 90% (18/20) in-hospital survival rate, while the autoantibody negative samples showed a 60% survival rate (9/15). Similarly, the autoantibody positive sepsis patients showed a 67% (4/6) survival rate and the autoantibody negative sepsis patients had a 71% (5/7) survival rate. Statistical analysis using Fischer's exact tests did not reveal any significant differences between the different groups. Lastly, this study with short-term samples from ARDS and sepsis patients was not designed to analyze the significance of these autoantibodies as they relate to long-term morbidity and mortality.

### Kinetics of autoantibody induction in ARDS and sepsis

**Figure 3:**
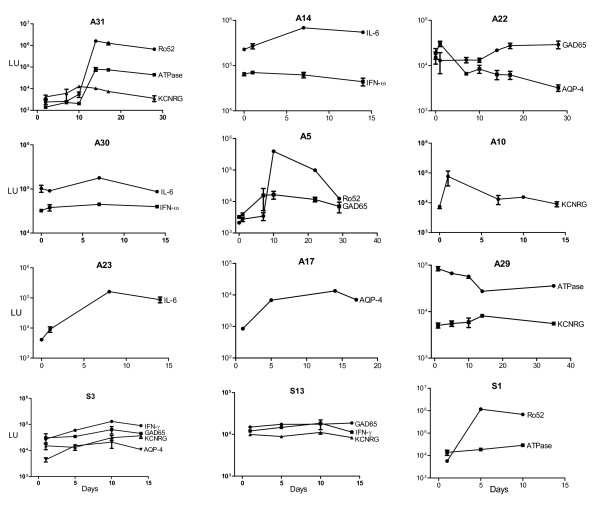
**Rapid and dynamic changes in autoantibody titer in ARDS and Sepsis patients**. Representative patient samples positive at day 10 for ARDS or day 14 for sepsis were reexamined for changes in antibody titers using all available serial samples. The antibody titers in LU plus standard error bars are plotted on the Y-axis using a log_10 _scale. The X-axis represents time in days following admission to the ICU.

Since 57% of the ARDS and 46% of the septic shock patients showed high antibody titers against at least one autoantigen, we analyzed serial samples to determine whether these were pre-existing antibodies or were generated during the acute inflammatory process. Available longitudinal samples, typically 3-5 different samples starting within the first two days after admission to the ICU were analyzed. Analysis of the ARDS autoantibody positive patients revealed dynamic changes in antibody titers over time. In some cases, the induced autoantibody titers showed a marked increase of 50 to 100-fold over the course of a few days (Figure 3). For example, the anti-Ro52 autoantibodies in ARDS patient A31 increased from 1,000 LU at day 10 to over 1 million LU by day 14 (Figure 3). A similar rapid rise in anti-ATPase and anti-KCNRG autoantibodies was also seen in patient A31 (Figure 3). Another patient (A5) showed a rapid rise in anti-Ro52 and to lesser extent anti-GAD65 autoantibodies, between days 1 and 10 after ICU admission (Figure 3). For patient A23, there was a dramatic rise in anti-IL-6 autoantibodies between day 0 and day 8 (Figure 3). A number of other patients including A14, A22, A10, and A17 showed autoantibody titer increases over time (Figure 3). Other patients, however, displayed high antibody titers from the beginning of their ICU admission, but showed an upward increase in antibody titers that peaked at day 8 (see the IL-6 serial titers inpatient A30; Figure 3). Lastly, some of these elevated autoantibodies remained high at the last serum sample collected at later time points such as at days 20 and 28 (Figure 3). Similar autoantibody titer increases and fluctuations were also seen in many of the sepsis patients (Figure 3). These results strongly suggest that autoantibodies can be rapidly induced and can markedly fluctuate during conditions of severe inflammation and/or infection such as ARDS and sepsis.

## Discussion

Our findings document the relatively high prevalence of autoantibodies in acute, inflammatory, high mortality conditions of ARDS and severe sepsis. The high detection rate of autoantibodies, 57% in ARDS and 46% in severe sepsis patients using a relatively small panel of autoantigens, suggests that the observed immunoreactivity to self proteins is a relatively common phenomenon in these two conditions. The most frequent autoantigen target in ARDS and sepsis was KCNRG, a protein highly expressed in the lung [20]. While autoantibodies to KCNRG have only been previously reported in a subset of autoimmune polyendocrine syndrome patients with lung complications [20], our finding of anti-KCNRG autoantibodies in ARDS and sepsis patients is consistent with the pulmonary injury and tissue destruction associated with these conditions. The detection of autoantibodies to the gastric ATPase autoantigen, a frequent target in a number of autoimmune conditions including autoimmune gastritis [21], type I diabetes [22] and Sjögren's syndrome [16], suggests that the stomach may be a highly promiscuous target of autoantibody attack in diverse inflammatory and autoimmune conditions. It should also be noted that many of the patients were concurrently on corticosteroids, but did not appear to block autoantibody production. The finding of the rapid induction of autoantibodies against the Ro52 autoantigen, one of the major rheumatological antigens comprising the SSA test, may coincide with the massive increase in antibodies directed at potential pathogens and human autoantigens that occur during ARDS and sepsis. Recent studies suggest that Ro52 autoantigen plays an important role in quality control of misfolded immunoglobulins produced by B-lymphocytes [23] and may be released from dying lymphocytes and other cells.

Consistent with the intense host inflammatory response found in ARDS (5) and sepsis [3], high titer autoantibodies were detected to a number of cytokines including IL-6, interferon-ω, interferon-γ and interleukin-1-α. In contrast to a previous report [24], we were unable to detect autoantibodies to IL-8 in any of the samples. Nevertheless, the finding that some patients show autoantibodies to a number of cytokines suggests that these antibodies may be biomarkers for the high levels of cytokines which may cause autoimmunization and possibly contribute to immune dysfunction seen in ARDS and sepsis. Alternatively these autoantibodies may play a role patient susceptibility to opportunistic infection. For example, anti-INF-γ autoantibodies are found in patients with susceptibility to non-tuberculosis mycobacterium infection [25-27], anti-IL-6 has been reported in a patient with chronic skin infection [28], and a variety of anti-cytokine autoantibodies are detected in a subset of thymoma patients with opportunistic infections [18,29,30]. Interestingly, 1 sepsis and 3 ARDS patients had relatively high titer autoantibodies against IL-6 and/or INF-ω suggesting that these autoantibodies might have a role in dampening the activity of these cytokines. Future investigations using other bioassays, such as looking for cytokine neutralizing activity, are necessary to further understand the functional significance of anti-cytokine autoantibodies in ARDS and sepsis.

Many of the autoantibody responses detected in ARDS and severe sepsis patients showed dynamic responses and marked changes in titer over a short period of time. Overall the findings of the rapid induction of autoantibodies against one or several autoantigen targets in the same patients do not support a role of molecular mimicry in inducing these antibodies. The mechanism for the rapid production of autoantibodies is intriguing. Long-term memory B-cells which are responsible for the extraordinary longevity of human serological memory [31] may also be involved in the rapid synthesis of autoantibodies described here. Rather than the long-term memory B-cells directed against pathogen proteins, small numbers of memory B-cells directed against self proteins may be present in all humans, but in most cases remain dormant. Following re-exposure to these self-antigens from tissue destruction and/or other antigen-independent mechanism including activation of cytokines and toll receptors, these memory B-cells may expand and differentiate into autoantibody producing plasma cells. Consistent with this notion is the finding that many of the autoantibody titers peaked at days 7-14 which may correlate with the time frame needed to induce these autoantibodies after the start of the inflammatory host response. Lastly, the time course for the rapid induction of autoantibodies seen in ARDS and sepsis may occur in other conditions including autoimmune diseases.

Although this study focused on short-term outcomes, it is intriguing that at these early time points autoantibodies associated with neurological targets are detected. There is evidence suggesting that ARDS patients suffer long-term adverse neuromuscular sequelae [32], and it is possible that autoantibodies and T-cell-mediated autoimmunity might contribute to these problems at later time points. For example, the presence of autoantibodies against AQP-4 and GAD65 in some ARDS and sepsis patients may be related to long-term neurological deficits seen in these patients. Anti-AQP-4 autoantibodies are found in patients with neurological complications including autoimmune attack on the optic nerve, spinal cord and peripheral nerves [16,33,34]. Anti-GAD65 autoantibodies have also been reported in a number of different neurological diseases including Stiff person syndrome, encephalitis and epilepsy, as well as being the major autoantigen in type I diabetes [35]. It is possible that the anti-AQP-4 and anti-GAD65 autoantibodies reflect autoimmune attack on the nervous system triggered by these conditions. Consistent with this possibility, it is interesting to note that some of autoantibodies detected in ARDS including to KCNRG, AQP-4 and GAD65, show sustained elevation past the last collected plasma samples at day 20 to 28. Since we were unable to analyze long-term outcome of these patients, it is unclear whether the presence of these autoantibodies are associated with long-term sequelae of critical illness. It is also unclear whether subsequent mild infections, inflammation and other trauma might reactivate autoantibody production at a later time in certain seropositive patients. Future studies expanding the autoantigen panel, profiling later time points and attempting to correlate autoantibody elevation with relevant clinical outcomes are needed to understand whether these autoantibodies have pathophysiological consequences.

## Competing interests

The authors declare that they have no competing interests.

## Authors' contributions

PDB, NS, SG and AFS conceived of the study. GUM collected the patient plasma samples and provided the clinical characteristics. PDB, SG, KHC and BH analyzed the sera by LIPS. PDB, NS and AFS analyzed the data. PDB drafted the manuscript. PDB, NS, MJI, GUM and AFS were involved in critical revision and final approval. All authors read and approved the manuscript.
